# Serratus Anterior Plane Block in the Emergency Department: A Case Series

**DOI:** 10.5811/cpcem.2019.11.44946

**Published:** 2020-01-21

**Authors:** Judy Lin, Taryn Hoffman, Ksenya Badashova, Sergey Motov, Lawrence Haines

**Affiliations:** Maimonides Medical Center, Department of Emergency Medicine, Brooklyn, New York

## Abstract

This is a case series of six emergency department (ED) patients who received an ultrasound-guided serratus anterior plane block (SAPB) for a variety of painful conditions. Our cases illustrate the feasibility and analgesic efficacy of the SAPB in providing pain management in ED patients with a variety of painful syndromes, including those with severe pain from multiple rib fractures, herpes zoster, and tube thoracostomy placement. In addition, we found no adverse events in our case series.

## INTRODUCTION

The serratus anterior plane block (SAPB) is a relatively new compartment block described in the anesthesia literature for the treatment of thoracic wall pain.^1^–^4^ Numerous studies have demonstrated the efficacy of SAPB for post-thoracotomy and post-mastectomy pain.^3^,^5^,^6^ However, of greater interest to emergency physicians (EP) is its use in patients with severe thoracic wall pain for whom opioids or non-steroidal anti-inflammatory drugs may not be an ideal treatment modality.

**Image 1:**
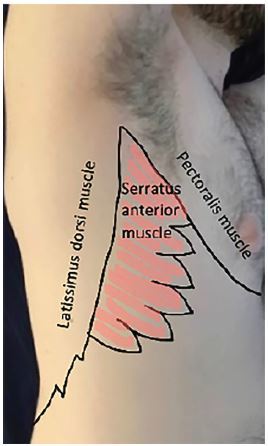
Serratus anterior plane block external landmarks.

**Image 2:**
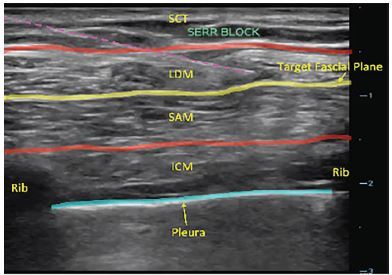
Serratus anterior plane block sono-anatomy. Yellow line, target plane; purple-dotted line, needle; blue line, pleura. *SCT*, subcutaneous tissue; *SAM*, serratus anterior muscle; *LDM*, latissimus dorsi muscle; *ICM*, Intercostal muscle.

The SAPB is a sensory nerve block that provides analgesia to the hemithorax from second thoracic (T2) to T9 dermatomes. Anesthetic, usually 25–30 milliliters (mL), is injected under ultrasound-guidance into the serratus anterior plane, either superficial or deep to the serratus anterior muscle (SAM). Within this fascial plane are the lateral cutaneous branches of the thoracic intercostal nerves.^1^,^3^ Image 1 depicts the external landmarks used to identify the proper injection site for the nerve block. Image 2 reveals the sono-anatomy of the serratus anterior plane and surrounding structures. The video demonstrates the SAPB being performed.

The most common indication for SAPB in the emergency department (ED) is for treatment of rib fracture pain. However, we also report a novel ED indication including treatment of herpes zoster pain and periprocedural pain from tube thoracostomy. Contraindications include local infection or allergy to the local anesthetic. Complications may result in local anesthetic toxicity, pneumothorax, and block failure.^1^,^7^

Patients with rib fractures have an increased risk of developing pneumonia, respiratory failure, and death, with mortality ranging from 4–20%.^8^–^10^ Treatment of such pain with opioids, especially in the elderly population, can lead to delirium and respiratory depression.^11^ Epidural and paravertebral blocks have been shown to decrease the risk of delirium and opioid requirements in patients with rib fractures, but require a patient to be sitting, lateral recumbent, or in a prone position.^11^,^12^ The SAPB can be performed in a supine patient and is, therefore, a more feasible alternative in the traumatic ED patient.

The evidence supporting the use of SAPB in the ED is limited to one prior case report in two ED patients for rib fracture pain.^13^ Additionally, there are no case reports describing EP-performed SAPB for tube thoracostomy and thoracic herpes zoster pain, although a few reports exist in the anesthesia literature.^14^,^15^ Here we present a case series of ED patients who received an EP-performed SAPB, and describe the extent of their injuries, the indication for the SAPB, the efficacy of the nerve block, and whether any adverse events due to the SAPB occurred during their hospital stay.

## CASE SERIES

### Case 1

An 85-year-old female with a medical history of meningioma, seizures, and hypertension, presented to the ED after a ground-level fall. On physical examination, the patient was uncomfortable and tender to her right flank. Chest radiography (CXR) and computed tomography (CT) demonstrated six posterior rib fractures (T4 to T9). The patient had pain with an intensity rating of 10/10, and reported minimal improvement despite administration of intravenous (IV) morphine sulfate at 8 milligrams (mg). The SAPB was performed using 30 mL of 0.25% bupivacaine deposited superficial to the SAM, with complete relief of pain 10 minutes after the block. The patient did not require any further pain medication in the ED, and received only acetaminophen during her inpatient stay. She was discharged without any use of opioids.

**CPC-EM Capsule**

What do we already know about this clinical entity?

One prior case series found that emergency physician (EP)-performed serratus anterior plane block (SAPB) can provide analgesia for rib fracture pain.

What makes this presentation of disease reportable?

There is limited literature describing the effects of EP-performed SAPB on a range of different, painful thoracic processes.

What is the major learning point?

SAPB can be an effective analgesic modality for thoracic diseases and injuries including rib fractures, herpes zoster, and thoracostomy placement.

How might this improve emergency medicine practice?

EPs may decide to use SAPB as a method for pain control, especially in patients for whom nonsteroidal anti-inflammatory drugs or opioids may have suboptimal side effects.

### Case 2

A 72-year-old male with medical history of diabetes and hypertension, and daily use of ticagrelor presented to the ED after a fall from standing that day. On physical examination, he was not in any respiratory distress and had tenderness to the left lateral chest wall. CXR showed displaced overriding, left-sided posterolateral fractures of ribs 8 to 11 that was confirmed on CT. SAPB was performed using 30 mL of 0.25% bupivacaine deposited superficial to the SAM. No other analgesics besides the SAPB were given. The patient reported complete pain relief after the procedure, required no further pain medication in the ED, and was admitted to the trauma service.

### Case 3

A 78-year-old female with a history of diabetes and hypertension presented to the ED with six days of “pimples” to her right chest with pain intensity rating of 10/10. On physical examination she had a classic zoster rash localized to lateral chest within the distribution of T5 to T8 dermatomes. The patient had a SAPB performed with 20 mL of 0.5% bupivacaine mixed with 60 mg of solumedrol deposited superficial to the SAM. The patient did not require any other pain medicine during her ED stay and reported significant pain relief. She was discharged on gabapentin 100 mg three times per day upon discharge, and did not have any return visits to the ED.

### Case 4

An 89-year-old female with history of atrial fibrillation on aspirin, hypertension, congestive heart failure (CHF), and chronic obstructive pulmonary disease presented to the ED, after being found on the floor by family, complaining of left-sided chest wall pain. On physical examination, the patient was tachypneic with a respiratory rate of 25 breaths per minute, and had left lateral chest wall tenderness. She was placed on bi-level positive airway pressure (BIPAP). Her CXR demonstrated left-sided rib fractures of indeterminate age with prominence of interstitial lung markings bilaterally. Her CT was remarkable for left-sided rib fractures 2 to 11 with a segmental fracture of rib seven and trace left-pleural effusion. Her venous blood gas showed a pH of 7.29 (7.320–7.430) and PCO2 of 71 millimeters of mercury (mm Hg) (38–50 mm Hg).

She and the family did not want intubation. Despite administration of 650 mg of oral acetaminophen, 10 mg of IV ketorolac, and 25 micrograms of IV fentanyl, the patient continued to complain of severe pain. There was concern that more opioids would worsen her sedation, hypercarbia and respiratory acidosis. Thus, the SAPB was performed with 30 mL of 0.25% bupivacaine with epinephrine deposited deep to the SAM. After the procedure, the patient reported numbness to her left chest wall and complete relief of her pain. She was discharged two days later with a prescription for acetaminophen and lidocaine patch.

### Case 5

A 94-year-old female with history of CHF and atrial fibrillation not on anticoagulation presented to the ED in respiratory distress. On arrival, her respiratory rate was 32 breaths per minute and oxygen saturation was 94% on non-rebreather. Her physical examination was significant for a chronically ill-appearing female in respiratory distress with decreased bibasilar breath sounds and poor air movement. Her B-type natriuretic peptide was 1180 picograms (pg) per mL (<100 pg/mL) and CXR showed new bilateral moderate-sized pleural effusions. She was placed on BIPAP without improvement. The patient and family did not want intubation, so the decision was made to insert a pigtail catheter for drainage of the pleural effusion. Pre-procedurally, a SAPB was performed using 30 mL of 0.25% bupivacaine deposited deep to the SAM. The patient tolerated the procedure well and did not require any pain medication besides the SAPB in the ED.

### Case 6

A 60-year-old female with no past medical history presented to the ED with severe, left-sided chest pain two days after a mechanical fall down a flight of stairs. She went to an urgent care center and had a CXR showing five displaced rib fractures and was sent to the ED. On arrival to the ED, the patient was tachypneic with a respiratory rate of 25 breaths per minute. On physical examination, she was uncomfortable appearing with tenderness to palpation over the left lateral ribs. In the ED she was given 4 mg of IV morphine but still could not tolerate lying flat for a chest CT due to worsening pain. A SAPB was performed with 20 mL of 0.25% bupivacaine deposited superficial to the SAM. The patient had significant relief of pain and was able to lie flat almost immediately after the block, which allowed her to tolerate lying supine for a CT that showed anterior rib fractures T5 to T9 and posterior rib fractures T8 to T10. The patient was admitted to the trauma service.

## DISCUSSION

Our case series reports on the positive efficacy of the SAPB performed in a series of six patients in the ED either as an alternative or as an adjunct to opioid and non-opioid analgesia. There was no evidence of SAPB-related adverse events.

SAPB was most commonly performed for rib fractures in our case series. Effective analgesia in patients with rib fractures is necessary for the prevention of reduced inspiratory volume and pneumonia.^9^,^12^ As illustrated in our descriptions, several patients could not achieve adequate pain control with parenteral opioids, but had pain relief with the block. A case series by Durant et al. also found similar improvement in rib fracture pain with the SAPB.^13^

SAPB was also performed for pain control in the treatment of acute herpes zoster pain as well as for procedural pain, ie, from tube thoracostomy, which has not been previously reported in the ED literature, although a few case reports in the anesthesiology literature exist.^14^,^15^ In our case series, patients had significant pain relief from the EP performed SAPB for both tube thoracostomy and acute herpes zoster. Our study included patients with anesthetic directed both superficial and deep to the SAM. Blanco et al. described similar spread of anesthesia as evidenced on magnetic resonance imaging for both locations relative to the SAM.^1^ Volunteers in Blanco’s study also showed similar distribution of chest wall analgesia whether they received superficial or deep injections.^1^ Our report indicates that both superficial and deep locations of anesthetic injections can result in effective analgesia.

This case series is limited due to its small size, and as this was not a retrospective chart review, data was not systematically collected. There is the possibility that the physician may not have documented all side effects. These findings do not evaluate the efficacy of the nerve block for all patients and does not identify instances of failed SAPB. In addition, the findings from this case series may not be generalizable. The dosing of parenteral pain medication was not standardized prior to the administration of the SAPB and, therefore, patients may have been underdosed, which could have exaggerated the impact of the nerve block on pain relief. All patients, except those from Cases 2 and 5, received an SAPB that was performed by an ultrasound fellowship-trained EP. The extra training and skill level required for performing this nerve block may limit the generalizability of this case series.

While this case series highlights the benefits of using SAPB in the patients described, further investigation must be pursued to determine the efficacy of and indications for all ED patients receiving a SAPB. Prospective studies evaluating the effectiveness of SAPB compared to traditional treatment for rib fracture pain, thoracostomy pain, and herpes zoster-related pain may further support the use of SAPB in these conditions.

## CONCLUSION

This case series found that SAPB was an effective treatment of pain secondary to rib fractures, herpes zoster, and tube thoracostomy with no adverse events. Based on our report, we believe that the SAPB can be an effective tool for the treatment of the aforementioned painful conditions and has several advantages over opioids that may make it an ideal treatment for specific patient populations. Further investigation is still needed to determine the role of SAPB in the management of a variety of patients with acutely painful conditions.

## Supplementary Information

Video. Serratus anterior plane block video. Yellow line = target plane; Purple-dotted line = needle.
*SAM*, serratus anterior muscle; *LDM*, latissimus dorsi muscle; *ICM*, intercostal muscle.
